# Value of a self-made subcutaneous negative-pressure drain in cesarean delivery: a propensity score-matched cohort study

**DOI:** 10.3389/fmed.2026.1882655

**Published:** 2026-09-17

**Authors:** Jie Cheng Bi, Ni Bei, Ying Liu, Kai Sun Zhao

**Affiliations:** Department of Obstetrics, The Third Affiliated Hospital of Guangxi Medical University, The Second Nanning People’s Hospital, Nanning, China

**Keywords:** cesarean delivery, negative-pressure drainage, propensity score matching, retrospective cohort study, wound complications

## Abstract

**Objective:**

To evaluate the preventive value of a self-made subcutaneous negative-pressure drain (5 mL or 10 mL syringe with a scalp vein needle) for wound complications after cesarean delivery.

**Methods:**

This retrospective cohort study included 259 women who underwent cesarean delivery (79 received the drain, 180 did not). Propensity score matching (1:1 nearest-neighbor, caliper = 0.2 SD) balanced baseline covariates (age, predelivery BMI, parity, prior surgeries, intrapartum transfer to cesarean, gestational age). After matching, 71 patients remained per group. The primary outcome was wound erythema/edema or fat liquefaction. Secondary outcomes included postoperative fever, length of stay, antibiotic duration, cost, and operative time. For binary outcomes, Fisher's exact test was used; for continuous variables, the Wilcoxon rank-sum test was used; multivariable linear regression was applied for adjusted analyses.

**Results:**

After matching, no baseline variable differed significantly between groups (all *P* > 0.05), although residual imbalance persisted for several variables (absolute standardized mean difference up to 0.359). The incidence of the primary outcome was 2.82% (2/71) in the study group vs. 5.63% (4/71) in controls (*P* = 0.681; OR 0.49, exact 95% CI 0.043–3.534). Postoperative fever was numerically more frequent in the study group (12.68% vs. 2.82%), but the difference was not statistically significant (Fisher's exact test, *P* = 0.055). Multivariable regression showed no significant effects on operative time (*β* = 1.75, *P* = 0.636), hospital stay (*β* = −0.11, *P* = 0.612), antibiotic duration (*β* = 4.34, *P* = 0.441), or cost (*β* = 1,621.44, *P* = 0.286).

**Conclusion:**

In this small retrospective cohort of an unselected cesarean population (not stratified by BMI or emergency/elective status), we found no evidence that the self-made subcutaneous negative-pressure drain reduces wound complications or improves other clinical/economic outcomes. Given the small number of events, the wide confidence intervals, and residual confounding, these findings are inconclusive: a clinically meaningful benefit or harm cannot be excluded, and routine use of this device cannot be recommended on the basis of the current evidence. Adequately powered prospective studies in predefined high-risk subgroups are needed.

## Introduction

Cesarean delivery is one of the most commonly performed major surgical procedures worldwide, and postoperative wound complications—including surgical site infection (SSI), fat liquefaction, seroma, and wound dehiscence—remain a major clinical challenge. SSI not only prolongs hospital stay, increases readmission rates, and raises healthcare costs, but is also associated with postpartum morbidity and maternal mortality (1, 2). Given the continuously rising global cesarean delivery rate (reaching 32% in the United States), optimizing wound management strategies to reduce the burden of complications has become a core priority for improving obstetric quality (1, 3). Negative-pressure wound therapy (NPWT) has recently been explored for the prophylactic management of cesarean incisions in high-risk populations. Single-use negative-pressure wound therapy (sNPWT) devices have shown theoretical advantages in high-risk obstetric patients, such as those with obesity, and several studies have compared their efficacy with that of standard dressings (4–6). However, the available evidence is inconsistent: some meta-analyses of randomized controlled trials suggest that prophylactic NPWT reduces the risk of SSI (5, 7), whereas individual randomized trials, including those conducted in high-risk (obese) populations, reported no significant benefit compared with conventional dressings (6, 8). Of note, most studies have focused on commercially available NPWT devices and have not adequately explored heterogeneity in device type, pressure parameters, or target populations (4, 9). Furthermore, the high cost of commercial devices limits their accessibility and feasibility in resource-limited settings. Subcutaneous drainage, as a mechanical preventive strategy, has been shown to reduce dead-space fluid accumulation and lower SSI risk in colorectal, gynecologic, and breast surgery (10, 11). Evidence from emergency laparotomy (e.g., for peritonitis) indicates that subcutaneous negative-pressure drainage significantly reduces the incidence of SSI, seroma, and wound exudate (10). In cesarean delivery specifically, the most directly relevant evidence concerns subcutaneous wound drains: a Cochrane review including ten randomized trials with 5,248 women found no evidence that routine wound drainage reduces the risk of wound infection, other wound complications, or febrile morbidity (12), and a meta-analysis of six randomized trials of prophylactic subcutaneous drainage likewise found no significant reduction in wound disruption, infection, hematoma, or seroma (13). A multicenter randomized trial in obese women with subcutaneous thickness of at least 4 cm also found no benefit of adding a subcutaneous drain to suture reapproximation (composite wound morbidity 22.7% vs. 17.4%; RR 1.3, 95% CI 0.8–2.1) (14). Systematic reviews point out that the evidence for the benefit of subcutaneous drainage in abdominal surgery is generally insufficient, and the necessity of routine prophylactic drainage has long been debated (15, 16). Specifically in the field of cesarean delivery, existing research has largely concentrated on commercial NPWT or standard drainage tubes, whereas rigorous evaluation of low-cost, simple self-made devices (e.g., a negative-pressure system constructed from a syringe and a scalp vein needle) is extremely scarce. Although favorable experiences with simplified negative-pressure systems in infected abdominal wounds have been reported, the efficacy and safety of their prophylactic use in cesarean incisions have not been rigorously examined in a real-world cohort.

This study therefore used a single-center retrospective design with propensity score matching (PSM) to systematically evaluate the preventive value of a self-made subcutaneous negative-pressure drainage device (a 5 mL or 10 mL syringe combined with a scalp vein needle) for wound complications after cesarean delivery. Unlike previous studies that focused on high-risk subgroups (e.g., obesity) or commercial devices (5, 7), our study enrolled a real-world population not stratified by BMI or by emergency/elective status, aiming to reflect routine clinical practice. We used wound erythema/edema or fat liquefaction as the primary early complication indicator and concurrently analyzed multidimensional outcomes including postoperative fever, length of hospital stay, healthcare resource consumption, and operative time. This study not only provides empirical evidence on the clinical applicability of a low-cost drainage strategy but also addresses the pressing need to balance “intervention necessity” with “resource appropriateness” in cesarean wound management, laying the groundwork for future prospective precision studies targeting high-risk subgroups (e.g., obesity, emergency surgery).

## Methods

### Study design and ethics

This was a single-center retrospective cohort study. The study protocol was approved by the Ethics Committee of the Second Nanning People's Hospital (approval number: Y2021013). Due to the retrospective nature of the study, the requirement for informed consent was waived.

### Study population

We included women who underwent cesarean delivery at the Second Nanning People's Hospital between February 2022 and November 2024. Inclusion criteria were: (1) gestational age ≥28 weeks; (2) delivery via a low transverse uterine incision; (3) both singleton and twin pregnancies were included. Exclusion criteria were: (1) pre-existing incision site infection or skin lesions; (2) severe systemic infection (e.g., sepsis); (3) intraoperative conversion to another surgical procedure (e.g., total hysterectomy); (4) severe anemia (hemoglobin <70 g/L); (5) incomplete clinical data. All women who received the device during the study period and had complete clinical records were included in the study group (79 cases); a small number of additional device recipients whose records were incomplete could not be included. Because the total number of cesarean deliveries performed at our hospital during the study period was not systematically recorded, the numbers of eligible women and of exclusions for each reason could not be quantified. For the control group, 180 women who underwent cesarean delivery during the same period without placement of the device were selected by the investigators from the delivery records; the control group therefore represents a convenience sample rather than a consecutive or exhaustive sample of all eligible controls.

### Exposure grouping

Women were divided into a study group (device placed) and a control group (no device) based on whether the self-made subcutaneous negative-pressure drainage device was placed during the operation. The device was prepared and placed as follows (Figure 1): a 5 mL or 10 mL disposable syringe was connected to the tubing of a scalp vein needle after removal of the metal needle, and 3–4 small holes (approximately 0.5 mm in diameter) were created in a staggered pattern on both side walls of the tubing. After evacuating air from the tubing, the distal end of the tubing was placed between the subcutaneous fat layer and the fascia, and the syringe plunger was pulled back to a predetermined volume (approximately 4 mL for the 5 mL syringe and 7 mL for the 10 mL syringe) and locked in place using the plunger clasp, creating a closed negative-pressure suction system. On the first postoperative morning, the attending surgeon assessed the plunger position and the integrity of negative-pressure maintenance during daily rounds; if the plunger had slipped, it was repositioned to the original volume and re-locked. The device was routinely maintained for 1–3 days. The indication for removal was a drainage volume of less than 2 mL over 24 h. No device-related severe complications (e.g., skin injury, active bleeding, or exacerbated local infection) were observed during the entire study period. The decision to place the device was made by the attending surgeon at the time of surgery on the basis of clinical judgment (e.g., perceived risk of wound complications related to subcutaneous fat thickness, anticipated hemostasis, or operator preference); no prespecified institutional protocol governed drain placement. Allocation therefore reflects routine clinical practice and is subject to confounding by indication. Of note, this device is not equivalent to regulated commercial closed-incision negative-pressure wound therapy (NPWT) systems: the negative pressure was not measured or standardized, two syringe sizes (5 mL and 10 mL) with different suction volumes were used, the drainage duration varied (1–3 days), and suction had to be restored manually when the plunger slipped.

**Figure 1:**
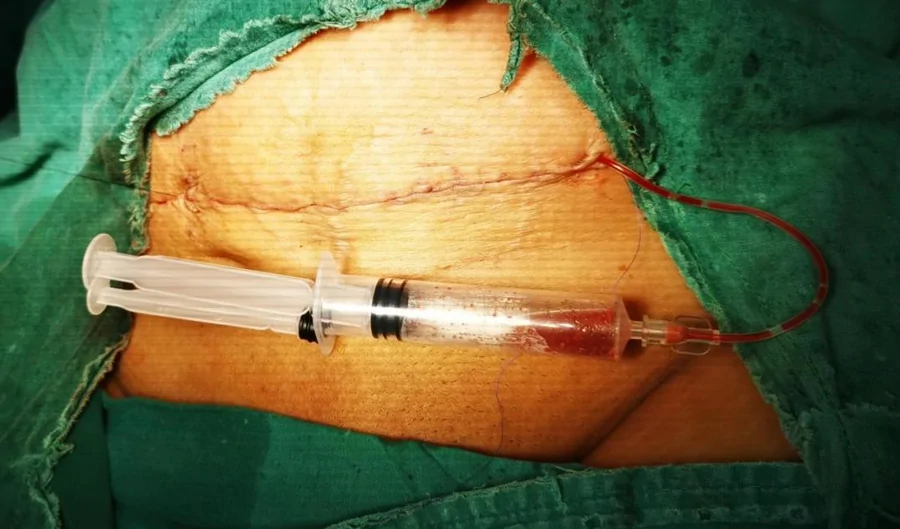
A simple negative-pressure suction system.

### Data collection

The following data were collected from the electronic medical record system:

Baseline data: age, gravidity, parity, number of prior surgeries (history of previous cesarean delivery), prepregnancy body mass index (BMI), predelivery BMI, gestational weight gain, history of assisted reproductive technology, and gestational age at delivery.

Perioperative indicators: preoperative and postoperative hemoglobin (Hb), white blood cell count (WBC), and neutrophil percentage (NEU); intraoperative blood loss; operative time (from skin incision to completion of subcuticular skin closure). Intrapartum transfer to cesarean (i.e., unplanned cesarean after a trial of labor) was also recorded.

### Outcome measures

Primary outcome: incisional erythema/edema or fat liquefaction (composite endpoint). Incisional erythema/edema was defined as redness and warmth of the skin around the incision, with or without tenderness; fat liquefaction was defined as yellow or clear serous exudate from the subcutaneous tissue, without purulent discharge.

Secondary outcomes: postoperative fever (postoperative fever was defined as an axillary temperature ≥37.3℃ on at least one occasion after surgery. Due to the retrospective nature of this study, we could not systematically distinguish the specific source of fever (e.g., endometritis, urinary tract infection, respiratory tract infection, or incisional infection) in all cases; therefore, this outcome was analyzed as a composite safety indicator rather than a wound-specific endpoint), postoperative length of hospital stay (from day of surgery to day of discharge), postoperative duration of antibiotic use (hours), and total hospitalization cost (in Chinese Yuan, CNY).

### Outcome assessment

Wound outcomes (incisional erythema/edema and fat liquefaction) and postoperative fever were ascertained from the electronic medical record during the index hospitalization, from the day of surgery until discharge; readmissions to our hospital for wound-related complications were also captured through the electronic medical record. No routine post-discharge wound assessment was performed, so wound complications that occurred after discharge and were managed at other institutions could not be captured. Because outcomes were extracted retrospectively from routine records, outcome assessors were not blinded to the exposure status.

### Statistical analysis

Statistical analyses were performed using SPSS 29.0 and R 4.3.3. To balance baseline differences between the two groups, PSM was employed. A logistic regression model was constructed with placement of the negative-pressure drain as the dependent variable, and the following covariates were included: age, predelivery BMI, parity, gestational age at delivery, number of prior surgeries, and intrapartum transfer to cesarean. The propensity score was calculated for each patient, and 1:1 nearest-neighbor matching was performed with a caliper of 0.2 standard deviations of the propensity score. After matching, 71 patients remained in each group. Before and after matching, continuous variables with a normal distribution were expressed as mean ± standard deviation (x¯ ± s), and between-group comparisons were performed using the independent-samples t-test. Continuous variables with a non-normal distribution were expressed as median (interquartile range) [M (Q_1_, Q_3_)], and between-group comparisons were performed using the rank-sum test. For matched binary outcomes, Fisher's exact test was used to provide conservative estimates given the small sample size. For unmatched comparisons, the *χ*^2^ test or Fisher's exact test (when expected frequencies were <5) was used as appropriate. For multivariable analysis, multivariable linear regression models were used for continuous outcomes (postoperative length of hospital stay, duration of antibiotic use, hospitalization cost, and operative time) in the matched sample. The covariates included in the models were parity, number of prior surgeries, intrapartum transfer to cesarean, age, predelivery BMI, and gestational age at delivery (all baseline variables; operative time was not adjusted for in the models for other outcomes to avoid mediator bias). For the binary outcomes (incisional erythema/edema or fat liquefaction, and postoperative fever), given the small number of events, only Fisher's exact test was used after matching; no multivariable logistic regression was performed. After matching, binary outcomes were compared as independent samples using Fisher's exact test. Because the pair identifiers generated by the 1:1 matching procedure were not retained in the analysis dataset, the original matched pairs could not be reliably reconstructed from the retrospective records, and an exact matched-pair analysis (e.g., McNemar's test) was therefore not feasible. For the primary outcome, the odds ratio and its 95% confidence interval (conditional exact [Fisher] method) were computed from the matched 2 × 2 table. The conditional exact interval inverts two one-sided exact tests based on the non-central hypergeometric distribution and is therefore consistent with the reported Fisher exact *P* value; no continuity correction and no large-sample (Woolf) approximation were applied. Multivariable linear regression was applied to continuous outcomes, several of which (hospitalization cost, duration of antibiotic use) were positively skewed; residual diagnostics were inspected, and these estimates should be interpreted with caution given the skewed distributions and the absence of matched-pair variance handling.

## Results

### Comparison of baseline characteristics between the two groups

Before matching, significant differences were observed between the study group and the control group in age, number of prior surgeries, prepregnancy BMI, and predelivery BMI (*P* < 0.05), with absolute SMD values exceeding 0.3 for these variables and for twin pregnancy, indicating baseline imbalance. The proportion of twin pregnancies did not differ significantly before matching (5.00% vs. 1.27%, *P* = 0.182, Fisher's exact test). After 1:1 propensity score matching (caliper = 0.2 standard deviations), 71 patients remained in each group. Although no baseline variable differed significantly after matching (*P* > 0.05), several variables retained nontrivial residual imbalance, with absolute SMD values of 0.359 (twin pregnancy), 0.292 (age), 0.255 (prepregnancy BMI), and 0.248 (predelivery BMI); the post-matching balance is therefore considered acceptable but imperfect, and baseline covariates were additionally adjusted in multivariable models. In the matched sample, twin pregnancies occurred in 4 cases (5.63%) in the control group and 1 case (1.41%) in the study group, with no significant difference (*P* = 0.366, Fisher's exact test; SMD 0.359) (Table 1).

**Table 1:** Comparison of baseline characteristics between the two groups.

Characteristic	Before PSM (*n* = 259)					After PSM (*n* = 142)				
	Control (*n* = 180)	Study (*n* = 79)	z/t/*χ*^2^	*P*	SMD	Control (*n* = 71)	Study (*n* = 71)	z/t/χ^2^	*P*	SMD
Age (years)	33 (28, 36)	35 (32, 37)	−3.143	0.002	0.518	36 (33, 38)	34 (32, 37)	−1.822	0.068	−0.292
Gestational age (days)	271 (265, 275)	269 (265, 274)	−1.410	0.158	−0.171	272 (264, 275)	270 (266, 274)	−0.196	0.845	−0.026
Gravidity	2 (2, 4)	2 (2, 4)	−0.341	0.733	0.049	3 (2, 4)	2 (2, 4)	−0.784	0.433	−0.100
Parity	2 (1, 2)	2 (1, 2)	−1.176	0.240	0.112	2 (1, 2)	2 (1, 2)	−0.791	0.429	−0.162
Number of prior surgeries	1 (1, 2)	2 (1, 2)	−2.386	0.017	0.316	2 (1, 2)	2 (1, 2)	−1.042	0.297	−0.177
Prepregnancy BMI (kg/m^2^)	22.59 (20.75, 25.00)	25.33 (22.83, 28.29)	−4.829	<0.001	0.618	24.29 (21.84, 26.59)	25.24 (22.52, 27.53)	−1.461	0.144	0.255
Predelivery BMI (kg/m^2^)	28.24 (26.04, 30.01)	31.24 (27.47, 33.22)	−5.017	<0.001	0.631	29.30 (27.40, 31.24)	31.22 (27.27, 32.98)	−1.699	0.089	0.248
Gestational weight gain (kg)	13.00 ± 4.45	12.60 ± 5.59	0.565	0.573	−0.072	12.95 ± 4.60	12.58 ± 5.19	0.455	0.650	−0.072
Assisted reproduction [*n* (%)]	33 (18.33%)	13 (16.46%)	0.133	0.716	0.051	16 (22.54%)	12 (16.90%)	0.712	0.399	0.150
Twin pregnancy [*n* (%)]	9 (5.00%)	1 (1.27%)		0.182^a^	−0.334	4 (5.63%)	1 (1.41%)		0.366^a^	−0.359

a Fisher's exact test.

BMI, body mass index.

### Comparison of laboratory indicators between the two groups

Before matching, there were no significant differences between the two groups in preoperative or postoperative hemoglobin, white blood cell count, or neutrophil percentage (*P* > 0.05). After matching, all laboratory indicators showed no statistically significant between-group differences (*P* > 0.05), with absolute SMD values <0.2, indicating balance in perioperative inflammation and anemia status between the two groups, (Table 2).

**Table 2:** Comparison of laboratory indicators between the two groups.

Laboratory indicator	Before PSM (*n* = 259)					After PSM (*n* = 142)				
	Control (*n* = 180)	Study (*n* = 79)	z/t/χ^2^	*P*	SMD	Control (*n* = 71)	Study (*n* = 71)	z/t/χ^2^	*P*	SMD
Preoperative Hb (g/L)	118.17 ± 11.58	120.00 ± 14.03	−1.098	0.273	0.131	119.48 ± 10.80	120.46 ± 14.41	−0.461	0.645	0.068
Postoperative Hb (g/L)	110 (100, 118)	112 (99.5, 119.5)	−0.581	0.561	0.010	110 (100, 118)	112 (99.5, 120)	−0.600	0.548	0.008
Preoperative WBC (×10^9^/L)	8.65 (7.47, 10.60)	8.90 (7.90, 10.15)	−0.515	0.606	0.010	8.30 (7.35, 10.05)	8.90 (7.90, 10.15)	−1.186	0.236	0.161
Postoperative WBC (×10^9^/L)	13.30 (11.35, 15.22)	12.80 (11.20, 15.55)	−0.140	0.889	−0.021	12.70 (10.80, 14.20)	12.80 (11.15, 15.50)	−1.039	0.299	0.185
Preoperative NEU (%)	73.65 (68.88, 76.85)	71.90 (68.15, 75.30)	−1.876	0.061	−0.216	72.11 ± 6.56	71.52 ± 5.77	0.568	0.571	−0.102
Postoperative NEU (%)	81.05 (77.60, 84.60)	80.60 (77.60, 83.05)	−0.768	0.442	−0.076	80.18 ± 5.91	80.32 ± 5.12	−0.149	0.882	0.027

Hb, hemoglobin; WBC, white blood cell; NEU, neutrophil percentage.

### Comparison of operative and outcome indicators between the two groups

Before matching, the study group had significantly longer operative time (65.0 vs. 60.5 min, *P* = 0.004), shorter postoperative hospital stay (4 vs. 5 d, *P* = 0.019), lower hospitalization cost (9,932.83 vs. 10,741.43 CNY, *P* = 0.021), and a higher rate of postoperative fever (11.39% vs. 3.89%; Fisher's exact test, *P* = 0.027). After matching, the study group had a lower median blood loss (300 vs. 400 mL, *P* = 0.048), shorter postoperative hospital stay (4 vs. 5 d, *P* = 0.029), lower hospitalization cost (9,932.83 vs. 10,973.63 CNY, *P* = 0.026), and a numerically higher rate of postoperative fever (12.68% vs. 2.82%) that did not reach statistical significance (Fisher's exact test, *P* = 0.055) than the control group. The differences in hospital stay and cost observed in univariable analyses were no longer statistically significant after multivariable adjustment (Table 4); whether these univariable differences reflect confounding, chance, or other factors cannot be determined from this study. No significant differences were found in operative time (*P* = 0.246), incidence of incisional erythema/edema or fat liquefaction (2/71 [2.82%] vs. 4/71 [5.63%], *P* = 0.681; OR 0.49, exact 95% CI 0.043–3.534), or duration of antibiotic use (*P* = 0.653) after matching (Table 3). No device-related severe complications (skin injury, active bleeding, or exacerbated local infection) were observed among the 71 patients in the study group.

**Table 3:** Comparison of operative and outcome indicators between the two groups.

Indicator	Before PSM (*n* = 259)					After PSM (*n* = 142)				
	Control (*n* = 180)	Study (*n* = 79)	z/t/χ²	*P*	SMD	Control (*n* = 71)	Study (*n* = 71)	z/t/χ²	*P*	SMD
Intrapartum transfer to cesarean [n(%)]	26 (14.44)	11 (13.92)	0.012	0.912	−0.015	7 (9.86)	11 (15.49)	1.018	0.313	0.156
Operative time (min)	60.5 (53.75, 72.00)	65.0 (57.50, 79.50)	−2.841	0.004	0.368	70 (60, 78.5)	65 (56.5, 77)	−1.161	0.246	0.022
Blood loss (mL)	400 (300, 400)	300 (300, 400)	−0.707	0.480	0.154	400 (300, 400)	300 (300, 400)	−1.981	0.048	0.073
Incisional erythema/edema or fat liquefaction [n(%)]	8 (4.44)	2 (2.53)		0.728^a^	−0.122	4 (5.63)	2 (2.82)		0.681^a^	−0.170
Postoperative fever [n(%)]	7 (3.89%)	9 (11.39%)		0.027^a^	0.236	2 (2.82%)	9 (12.68%)		0.055^a^	0.296
Postoperative hospital stay (d)	5 (4, 5)	4 (4, 5)	−2.352	0.019	−0.063	5 (4, 5)	4 (4, 5)	−2.181	0.029	−0.083
Duration of antibiotic use (h)	24 (24, 48)	24 (24, 24)	−1.298	0.194	0.030	24 (24, 48)	24 (24, 24)	−0.449	0.653	0.174
Hospitalization cost (CNY)	10,741.43 (9,642.02, 11,828.31)	9,932.83 (8,183.36, 12,072.36)	−2.312	0.021	0.124	10,973.63 (9,760.85, 11,734.60)	9,932.83 (8,075.53, 12,025.10)	−2.225	0.026	0.107

a Fisher's exact test. OR (95% CI) for incisional erythema/edema or fat liquefaction after matching (study vs. control): 0.49; exact (conditional) 95% CI: 0.043–3.534.

**Table 4:** Multivariable linear regression analysis: association between the self-made subcutaneous negative-pressure drainage device and surgical outcomes (adjusted).

Outcome	*β* (95% CI)	*P*
Operative time (min)	1.75 (−5.48–8.97)	0.636
Postoperative hospital stay (d)	−0.11 (−0.52–0.31)	0.612
Duration of antibiotic use (h)	4.34 (−6.66–15.34)	0.441
Hospitalization cost (CNY)	1,621.44 (−1,344.97–4,587.84)	0.286

Adjusted for parity, number of prior surgeries, intrapartum transfer to cesarean, age, predelivery BMI, and gestational age at delivery. Reference: control group.

### Adjustment for confounding factors

After adjusting for parity, number of prior surgeries, intrapartum transfer to cesarean, age, predelivery BMI, and gestational age at delivery, placement of the self-made subcutaneous negative-pressure drainage device had no statistically significant association with operative time (*β* = 1.75, 95% CI −5.48 to 8.97, *P* = 0.636), postoperative hospital stay (*β* = −0.11, 95% CI −0.52 to 0.31, *P* = 0.612), duration of antibiotic use (*β* = 4.34, 95% CI −6.66 to 15.34, *P* = 0.441), or hospitalization cost (*β* = 1,621.44, 95% CI −1,344.97 to 4,587.84, *P* = 0.286) (Table 4). Intraoperative blood loss was not included in the multivariable models because it was not a prespecified outcome and its distribution was highly skewed. The differences in hospital stay and cost observed in univariable analyses were not confirmed in the adjusted analyses; whether these univariable differences reflect confounding, chance, or other factors cannot be determined from this study.

## Discussion

In this propensity score-matched cohort analysis, we evaluated the value of a self-made subcutaneous negative-pressure drainage device (a 5 mL or 10 mL syringe combined with a scalp vein needle) in preventing wound complications after cesarean delivery. Among 142 matched women (71 per group), the device was not significantly associated with the incidence of wound erythema/edema or fat liquefaction (2/71 [2.82%] vs. 4/71 [5.63%], *P* = 0.681; OR 0.49, exact 95% CI 0.043–3.534), nor with the secondary outcomes of length of hospital stay, duration of antibiotic use, hospitalization cost, or operative time. Postoperative fever was numerically higher in the study group, but the difference was not statistically significant (12.68% vs. 2.82%, Fisher's exact test, *P* = 0.055). These findings provide no evidence of a prophylactic benefit of this simple device in this unselected population; however, given the small number of events and the correspondingly wide confidence interval (exact 95% CI 0.043–3.534, compatible both with a large reduction and with a more than threefold increase in the odds of the outcome), a clinically meaningful benefit or harm cannot be excluded.

### Comparison with previous evidence

Our findings are consistent with the most directly relevant evidence on subcutaneous drainage at cesarean delivery. A Cochrane review of ten randomized trials including 5,248 women found no evidence that the routine use of wound drains at cesarean delivery reduces the risk of wound infection, other wound complications, or febrile morbidity, and concluded that neither moderate benefit nor harm could be excluded (12). Similarly, a meta-analysis of six randomized trials of prophylactic subcutaneous drainage after cesarean delivery reported no significant reduction in wound disruption (OR 0.74, 95% CI 0.39–1.42), infection (OR 1.15, 95% CI 0.70–1.90), hematoma, or seroma (13), and the multicenter randomized trial by Ramsey et al., which enrolled obese women with subcutaneous thickness of at least 4 cm, found that adding a subcutaneous drain to suture reapproximation did not reduce composite wound morbidity (22.7% vs. 17.4%; RR 1.3, 95% CI 0.8–2.1) (14). By contrast, prophylactic negative-pressure wound therapy (NPWT) using regulated commercial devices has been reported to reduce SSI in some meta-analyses of randomized trials in high-risk (mainly obese) cesarean populations (5, 7), whereas individual randomized trials found no significant benefit (6, 8); the most recent Cochrane review of NPWT for primarily closed surgical wounds concluded that the existing evidence remains uncertain (17). Importantly, the self-made device evaluated here is not equivalent to these commercial NPWT systems: its negative pressure was not measured or standardized, suction was restored manually, and the drainage duration varied. Therefore, neither the positive nor the negative results obtained with commercial NPWT can be directly extrapolated to this device. Cesarean incision infection is a major global obstetric complication (1, 18), and its prevention requires a comprehensive approach encompassing surgical technique, antibiotic prophylaxis, and individual patient risk factors; the effect of any single intervention is inherently limited.

### Possible explanations for the higher postoperative fever rate

The numerically higher rate of postoperative fever observed in the study group (12.68% vs. 2.82%) was not statistically significant (Fisher's exact test, *P* = 0.055) and should be interpreted with caution. Due to the retrospective nature of this study, we could not reliably subdivide the etiology of fever (e.g., endometritis, urinary tract infection, pneumonia, or incisional infection) in all cases. Therefore, this finding cannot be directly attributed to the drainage device. The unexpected direction of this difference may reflect residual confounding, random variation due to small numbers, or unmeasured differences in baseline risk factors. Potential risks of the simple device, such as local irritation from the retained tubing, inadequate sealing with risk of retrograde infection, or compromised drainage due to plunger slippage, cannot be entirely excluded but remain speculative. This observation should be considered hypothesis-generating rather than a safety signal, and prospective studies with predefined fever workup protocols are needed to clarify this issue.

### Strengths and limitations

The strengths of this study include the use of propensity score matching to reduce, although not eliminate, imbalance in key observed confounders (e.g., age, BMI, parity, surgical history), and the assessment of outcomes across both clinical and economic dimensions, which aligns with real-world decision-making needs.

Several limitations should be acknowledged. The single-center retrospective design is subject to selection bias. In addition, the control group was selected as a convenience sample from contemporaneous cesarean deliveries without the device rather than representing a consecutive or exhaustive sample, and the total number of cesarean deliveries during the study period was not systematically recorded; the magnitude of any resulting selection bias therefore cannot be quantified. Although PSM adjusted for observable confounders, unmeasured confounders (e.g., operator habits, details of incision closure technique) may still influence the results. The overall sample size was limited, with only six events of the primary outcome (incisional erythema/edema or fat liquefaction), resulting in insufficient statistical power to detect small but clinically meaningful differences. The self-made device lacks standardized negative-pressure parameters (e.g., pressure value, drainage duration) and differs substantially from commercial NPWT systems; therefore, our findings should not be directly extrapolated to professional negative-pressure therapy devices. In addition, because the decision to place the drain was made by the attending surgeon, allocation was subject to confounding by indication; before matching, treated women were older, had higher BMI, and had more previous surgery, and several determinants of both drain use and wound complications (e.g., diabetes, chorioamnionitis, duration of membrane rupture, duration of labor, antibiotic prophylaxis, surgeon experience, closure technique, subcutaneous thickness) were not available for adjustment. Wound outcomes were ascertained during the index hospitalization (with readmissions to our hospital captured), but no standardized post-discharge surveillance was performed, so wound complications developing after discharge and managed elsewhere may have been missed. Postoperative fever was defined as a single axillary temperature ≥37.3°C without a standardized diagnostic workup and is a nonspecific indicator. Finally, matched binary outcomes were analyzed as independent samples because the pair identifiers generated by the 1:1 matching procedure were not retained in the analysis dataset and the original matched pairs could not be reconstructed from the retrospective records, which precluded an exact matched-pair analysis; in addition, linear regression was applied to several skewed continuous outcomes without matched-pair variance handling. Given the very small number of events, these analyses have limited power, and our data remain compatible with both benefit and harm. We also did not perform subgroup analyses based on high-risk categories such as BMI ≥30, emergency surgery, or diabetes mellitus.

Several important confounders could not be included in the PSM model due to the limitations of retrospective data extraction. These include diabetes mellitus, gestational diabetes, hypertensive disorders, smoking status, duration of labor, duration of membrane rupture, chorioamnionitis, surgeon experience, wound closure technique, and subcutaneous tissue thickness. While we adjusted for predelivery BMI (which partially reflects metabolic status) and gestational age at delivery (which is closely related to preterm-related infectious risk), we acknowledge that unmeasured confounding may have influenced the observed outcomes. This is a key limitation of our study and should be addressed in future prospective investigations. The limited sample size after PSM (71 patients per group) and the small number of primary outcome events (only 6 cases) increase the risk of type II error (i.e., failing to detect a clinically meaningful difference). With such a low event rate, even a moderate protective effect could have been missed. Therefore, our findings should be interpreted as exploratory rather than definitive. A future adequately powered prospective study (estimated ≥300 patients per group for binary outcomes with an event rate of ∼5%) is needed to confirm or refute the potential benefit of this device.

We did not perform subgroup analyses based on BMI (e.g., ≥30 kg/m^2^), diabetes mellitus, emergency vs. elective cesarean delivery, or prior cesarean section history. This was primarily due to the limited sample size after PSM; further stratification would have yielded very small subgroups (e.g., obese patients, *n* ≈ 15–20 per group) and unreliable estimates. Since most of the existing literature on prophylactic negative-pressure therapy has focused on high-risk populations (particularly obesity and diabetes), the absence of such subgroup analyses limits the generalizability of our findings to these specific groups. Our results apply primarily to an unselected, routine clinical population. We believe that future prospective studies with larger sample sizes and prespecified subgroup analyses are necessary to determine whether this low-cost device might offer benefit in selected high-risk patients.

## Conclusion

Based on this single-center retrospective cohort, in an unselected population of women undergoing cesarean delivery (i.e., not stratified by BMI or emergency/elective status), we found no evidence that the self-made subcutaneous negative-pressure drainage device reduces the incidence of wound erythema/edema or fat liquefaction or improves other clinical or economic outcomes. The numerically higher rate of postoperative fever in the study group was not statistically significant and should be interpreted with caution. Owing to the small sample size, the low number of events, the residual imbalance after matching, and the retrospective design, these findings are inconclusive: a clinically meaningful benefit or harm cannot be excluded. This study therefore does not provide evidence to support the routine preventive use of this device in cesarean delivery, and adequately powered prospective studies in predefined high-risk subgroups are needed.

## Data Availability

The original contributions presented in the study are included in the article/Supplementary Material, further inquiries can be directed to the corresponding author/s.
